# Kukoamine A inhibits human glioblastoma cell growth and migration through apoptosis induction and epithelial-mesenchymal transition attenuation

**DOI:** 10.1038/srep36543

**Published:** 2016-11-08

**Authors:** Qiaoping Wang, Haiyan Li, Zhen Sun, Lihua Dong, Ling Gao, Chunlan Liu, Xiujie Wang

**Affiliations:** 1 Laboratory of Experimental Oncology, State Key Laboratory of Biotherapy/Collaborative Innovation Center for Biotherapy, West China Hospital, West China Clinical Medical School, Sichuan University, Chengdu 610041 China

## Abstract

*Cortex lycii radicis* is the dried root bark of Lycium chinense, a traditional Chinese herb used in multiple ailments. The crude extract of *Cortex lycii radicis* has growth inhibition effect on GBM cells. Kukoamine A (KuA) is a spermine alkaloid derived from it. KuA possesses antioxidant, anti-inflammatory activities, but its anticancer activity is unknown. In this study, the growth and migration inhibition effect of KuA on human GBM cells and the possible mechanism of its activity were investigated. After KuA treatment, proliferation and colony formation of GBM cells were decreased significantly; apoptotic cells were increased; the cell cycle was arrested G0/G_1_ phase; the migration and invasion were decreased, the growth of tumors initiated from GBM cells was inhibited significantly; the expressions of 5-Lipoxygenase (5-LOX) were decreased, apoptotic proteins, Bax and caspase-3 were increased, and antiapoptotic protein Bcl-2 was decreased significantly; The expressions of CCAAT/enhancer binding protein β (C/EBPβ), N-cadherin, vimentin, twist and snail+slug were decreased significantly, while the expression of E-cadherin was increased significantly in KuA treated GBM cells and tumor tissues. KuA inhibited human glioblastoma cell growth and migration *in vitro* and *in vivo* through apoptosis induction and epithelial-mesenchymal transition attenuation by downregulating expressions of 5-LOX and C/EBPβ.

Glioblastoma (GBM) is the most common and malignant human primary brain tumor with poor prognosis^1^,^2^. Despite current progresses in therapeutic modalities for GBM, such as surgery, radiotherapy and chemotherapy, the outcome for GBM patients remains dismal; the recurrence is inevitable, invasive growth is a major cause of the high mortality of GBM^1^,^2^,^3^. The malignant tumor cells are characterized with unlimited proliferation, migration and invasion potential^4^; the diffuse invasion of GBM enables it to escape complete surgical resection and chemo- and radiation therapy, which is a major obstacle to eradicate GBM^4^,^5^. Moreover, glioblastomas are resistant to chemotherapy, radiation and other adjuvant therapies, no effective therapy is then available currently^6^,^7^. Therefore, there is an urgent need to research and develop more novel effective therapeutic options and increase the efficacy of radio/chemotherapy for this very aggressive and malignant brain tumor^6^,^7^,^8^,^9^.

The unique biological behavior of GBM invasion might provide so far unexplored brain-specific therapeutic targets for treatment of this lethal tumor^5^. Thus, inhibiting proliferation, migration and invasion of GBM cells are regarded as effective strategic modalities to screen a new drug for GBM treatment^10^,^11^,^12^,^13^,^14^. Phytochemicals derived from medicinal herbs and dietary plants have recently received much attention as potential therapeutic and preventive agents for cancers, including GBM^15^,^16^.

*Cortex lycii radicis* is the dried root bark of Lycium chinense, a traditional Chinese herb. It was generally used to treat lung fever, cold blood, help reduce blood pressure, etc^17^. The crude extract of *Cortex lycii radicis* has growth inhibition effect on GBM cells (U87MG)^16^.

Kukoamine A (KuA), a spermine alkaloid, is a major bioactive component in *Cortex lycii radicis.* It processes antihypertensive, antioxidant, anti-inflammatory, soybean lipoxygenase inhibition and neuroprotection activities^18^,^19^, and protects neuroblastoma SH-SY5Y cells from H_2_O_2_ induced oxidative stress damage^19^.

Lipoxygenase plays vital role in chronic inflammation and carcinogenesis^20^. 5-Lipoxygenase (5-LOX) exerts an enormous function in carcinogenesis, progression and prognosis of primary glioblastomas^21^. GBM expressed higher level of 5-Lipoxygenase (5-LOX) than low grade low-grade astrocytoma^22^. 5-LOX inhibition might be a candidate target therapy for patients with 5-LOX-expressing malignant gliomas. 5-lipoxygenase inhibitors exhibited potent growth inhibition effect on glioma cells *in vitro*^23^,^24^,^25^,^26^. So, exploring and developing novel lipoxygenase inhibitors may have an important role in cancer prevention and treatment, including GBM^27^.

In light of these findings, it could be postulated that KuA might have growth inhibition effect on human cancers, such as GBM. However, there is no such a study reported. In order to test this hypothesis, the present study was therefore designed to assess whether KuA could inhibit human GBM cell growth *in vitro* and *in vivo*, and to elucidate its potential mechanism of anticancer activity.

## Results

### KuA suppresses human GBM cell proliferation *in vitro*

**Figure 1:**
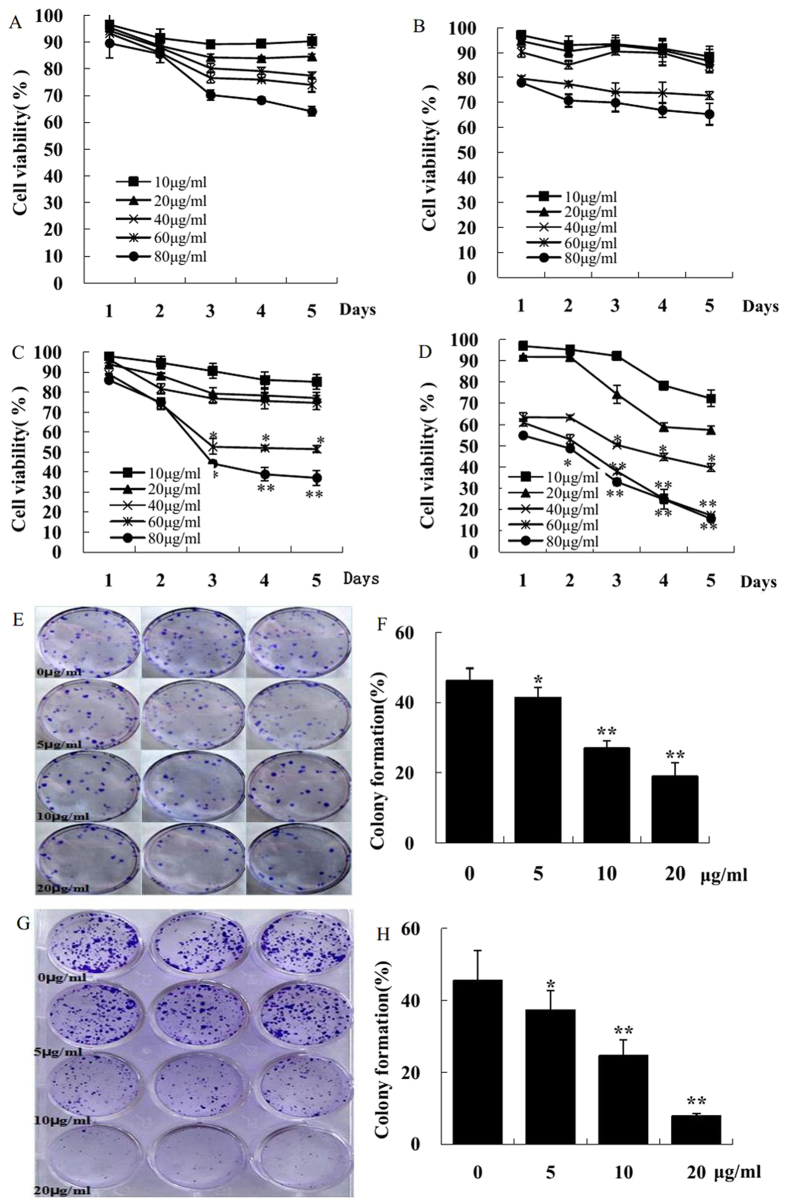
KuA inhibits human glioblastoma cell growth and colony formation *in vitro.* Normal cells and GBM cells were seeded onto 96-well plate at 1.5 × 10^3^/well and were treated with KuA at different concentrations, and percentage of cell viability was determined by MTT assay after 1d, 2, 3, 4, 5 days of treatment, respectively; GBM cells (U251 and WJ1) were seeded in 6 well plates at a density of 150 cells/well and 300 cells/well, the colony number was counted under dissection microscope. Results are mean values ± SEM of three independent experiments, *P < 0.05, **P < 0.01. (**A**) KuA inhibits human normal liver cell growth (L02). (**B**) KuA inhibits rat glioma cell growth (C6). (**C**) KuA inhibits human glioblastoma cell growth (U251). (**D**) KuA inhibits human glioblastoma cell growth (WJ1): (**E,F**) KuA inhibits human glioblastoma cell colony formation (U251) dose dependently. (**G,H**) KuA inhibits human glioblastoma cell colony formation (WJ1) dose dependently.

Growth inhibition effect of KuA on GBM cells and normal cells *in vitro* was determined by cytotoxicity assay (Fig. 1A–D). KuA exhibited a time and dose-dependent inhibitory effect on human GBM cells (*p* < 0.01). IC_50_ was 73.4 and 22.1 μg/mL for U251 and WJ1 cells, respectively; 226.0 and 217.0 μg/mL for rat glioma cells (C6) and human normal liver cells (LO2) at day 5. The maximal inhibition of human GBM cell growth (>80%) was obtained at 60–80 μg/mL. Together, the results demonstrate that KuA inhibited human GBM cell growth selectively *in intro*.

### KuA inhibited colony formation of human GBM cells

Cloning efficiency of untreated U251 and WJ1 cells were 46.2 ± 3.4% and 45.6 ± 8.2%, respectively. Colony formation was suppressed significantly in a dose-dependent manner after treated with 5, 10, and 20 μg/mL of KuA (Fig. 1E,H, *p* < 0.01) in both U251 and WJ1 GBM cells. These results suggest that KuA suppressed the clonogenicity of U251 and WJ1 cells (Fig. 1E–H, *p* < 0.01).

### KuA induced apoptotic cells detected by AO/EB staining and flow cytometry

**Figure 2:**
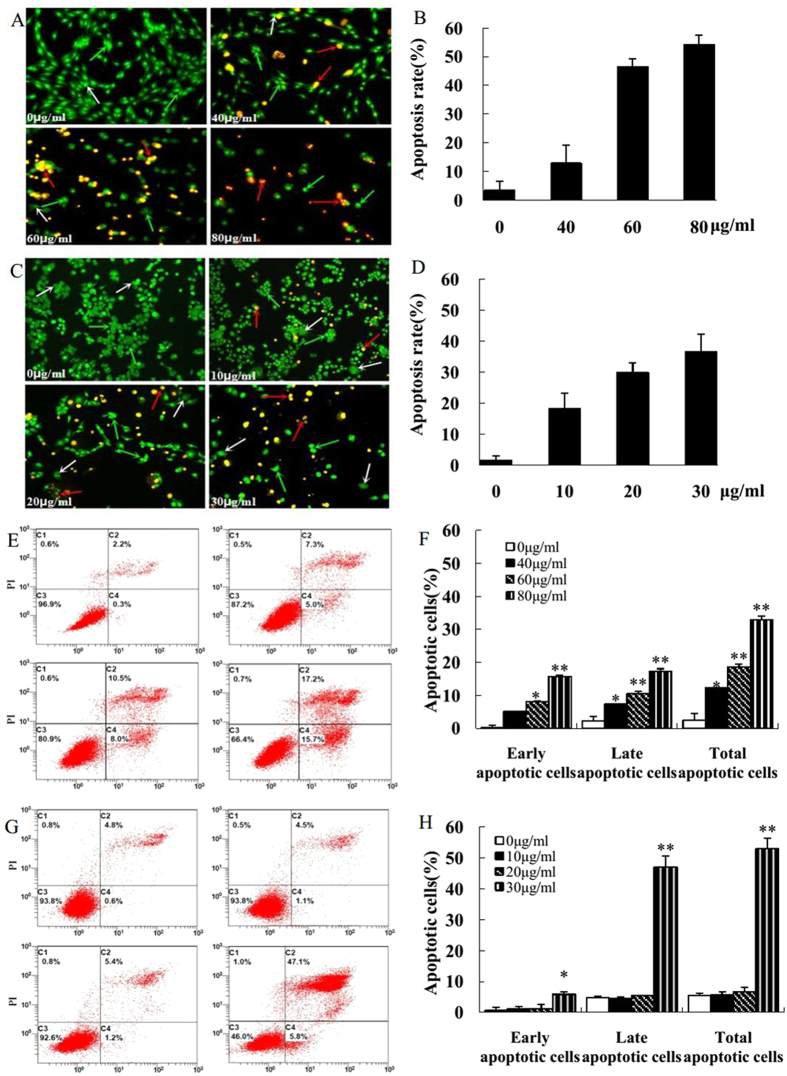
KuA induces apoptosis of human glioblastoma cells. Human GBM cells (U251and WJ1) were treated with different concentrations of KuA for 48 h. Apoptotic cells were detected with AO/EB staining and flow cytometry analysis (Annexin V-FITC/PI double staining). Results are mean values ± SEM of three independent experiments, *P < 0.05, **P < 0.01. (**A**) AO/EB stained apoptotic cells in KuA treated U251 cells. (**B**) The histogram shows that there was significant increase of AO/EB stained apoptotic cells in KuA treated U251 cells. (**C**) AO/EB stained apoptotic cells in KuA treated WJ1 cells. (**D**) The histogram shows that there was significant increase of AO/EB stained apoptotic cells in KuA treated WJ1 cells. The green arrow indicates live cells. The white arrow indicates early apoptotic cells, red arrow later apoptotic cells. (**E**) KuA induces apoptosis of U251 cells. (**F**) The histogram shows the significant increase of apoptotic cells in U251 cells after treatment with KuA in a dose dependent manner. (**G**) KuA induces apoptosis of WJ1 cells. (**H**) The histogram shows the significant increase of apoptotic cells in WJ1 cells after treatment with KuA in a dose dependent manner.

KuA induced apoptotic cells were detected by AO/EB staining and flow cytometry. By AO/EB staining, the apoptotic cells in untreated U251 and WJ1 cells were 3.5 ± 2.4% and 1.6 ± 1.3%, respectively. After treatment with 40, 60 and 80 μg/ml of KuA for U251 cells and 10, 20 and 30 μg/ml of KuA for WJ1 cells for 48 h, respectively, the apoptotic cells in treated U251 and WJ1 cells increased significantly in a dose dependent manner (Fig. 2A–D, *p* < 0.01); Annexin-V-FITC/PI double staining assay and FCM analyses showed that apoptotic cells in the untreated U251 and WJ1 cells were 2.5 ± 1.9% and 5.4 ± 0.7%, respectively, After treatment with 40, 60 and 80 μg/ml of KuA for U251 cells and 10, 20 and 30 μg/ml of KuA for WJ1 cells 48 h, respectively, the apoptotic cells in the treated U251 and WJ1 cells increased significantly in a dose dependent manner increased significantly in a dose-dependent manner (Fig. 2E–H, *p* < 0.01). These results indicate that KuA induced apoptosis of U251 and WJ1 cells.

### KuA induced cell cycle arrest of GBM cells

**Figure 3:**
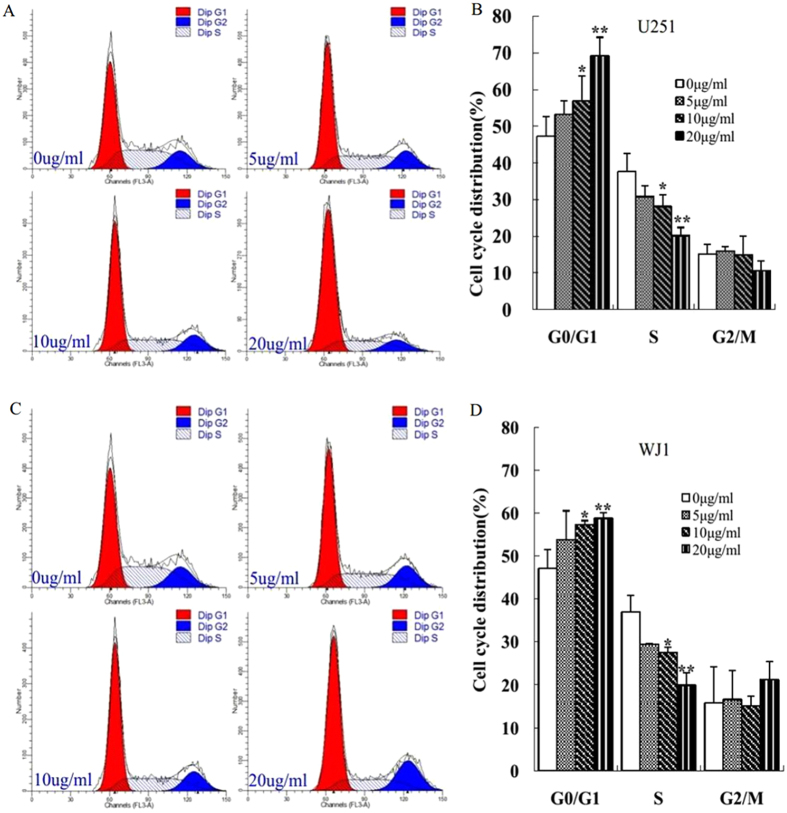
KuA induces cell cycle arrest of human GBM cells. Human GBM cells (U251and WJ1) were treated with different concentrations of KuA for 48 h. both the control and treated cells were harvested, stained with 0.05% propidium iodide and subjected to flow cytometric analysis. Results are mean values ± SEM of three independent experiments, *P < 0.05, **P < 0.01. (**A**) KuA induces cell cycle arrest of U251 cells. (**B**) The histogram shows the significant increase of cell population in G_0_/G_1_ phase and decrease in S phase in a dose dependent manner in KuA treated U251cells (**C**) KuA induces cell cycle arrest of WJ1 cells. (**D**) The histogram shows the significant increase of cell population in G_0_/G_1_ phase and decrease in S phase in a dose dependent manner in KuA treated WJ1 cells.

Cell cycle analysis was performed in KuA treated U251 and WJ1 cells. After KuA treatment, the cell population in G_0_/G_1_ phase was increased and decreased in S phase in a dose dependent manner in both U251 and WJ1 cells (Fig. 3A–D). These results demonstrate that KuA treatment caused U251 and WJ1 cells cell cycle arrest in G_0_/G_1_ phase.

### KuA inhibited GBM cell migration

**Figure 4:**
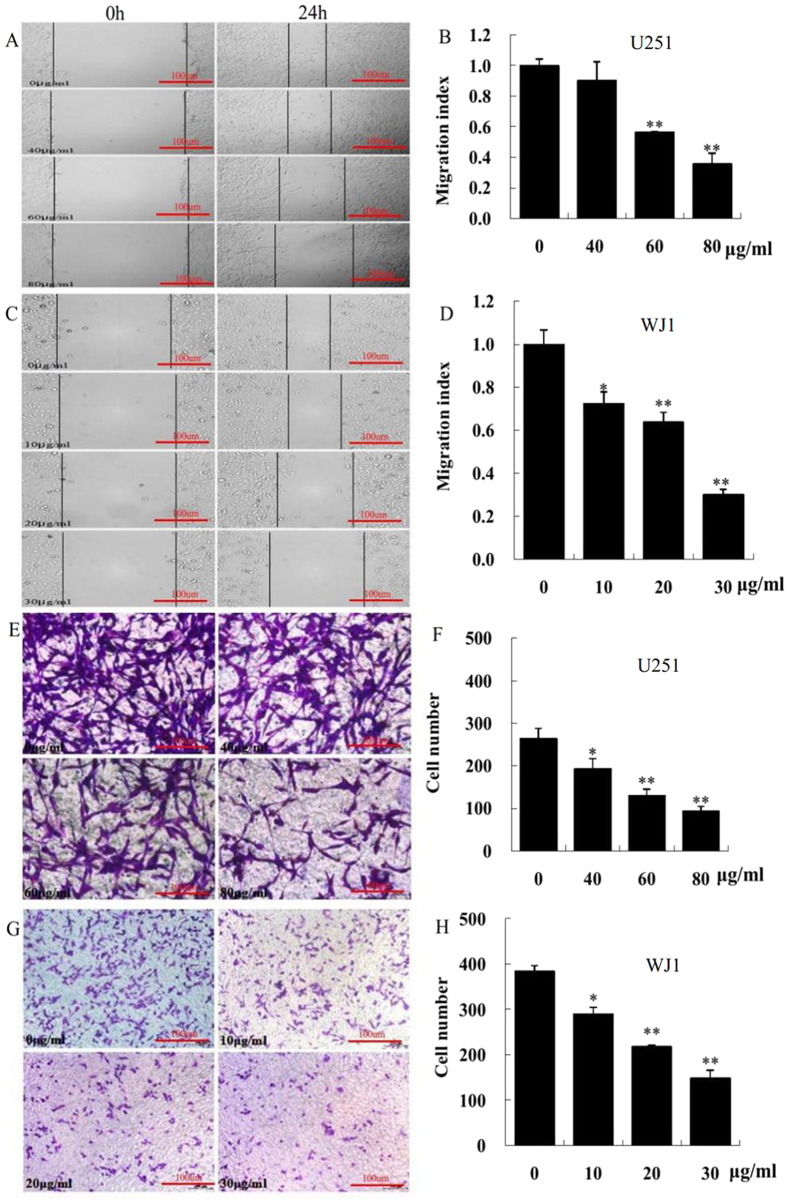
KuA inhibits human glioblastoma cell migration and invasion. Human GBM cells (U251and WJ1) were treated with different concentrations of KuA for 24 h. Wounds were made with a 10 μL pipette tip, Photographs were taken immediately (time 0) and 24 h after wounding for GBM cells, The distances migrated by the monolayer cell to close the wounded area were measured; Cell invasion assay was performed in 24-transwell chambers, the cells that penetrated through the filter were counted. Results are mean values ± SEM of three independent experiments, *P < 0.05, **P < 0.01. (**A**) KuA inhibits U251 cell migration. (**B**) The histogram shows that KuA inhibits U251 cell migration dose-dependently. (**C**) KuA inhibits WJ1 cell migration. (**D**) The histogram shows that KuA inhibits U251 cell migration dose-dependently. (**E**) KuA inhibits U251 cell migration invasion. (**F**) The histogram shows that KuA inhibits U251 cell invasion dose-dependently. (**G**) KuA inhibitsWJ1 cell migration invasion. (**H**) The histogram shows that KuA inhibitsWJ1 cell invasion dose-dependently.

The distance moved by the untreated and KuA treated U251 and WJ1 monolayer cells was measured and the results were expressed as migration indexes, which represent the distance migrated by KuA-treated cells relative to the distance migrated by the untreated cells. The migration abilities of KuA-treated U251 and WJ1 cells were significantly decreased in a dose-dependent manner (Fig. 4A–D, *p* < 0.01). Together, these results indicate that KuA inhibited migration of U251 and WJ1 cells.

### KuA inhibited GBM cell invasion

The cell numbers penetrated the transwell membrane of the untreated U251 and WJ1 cells were 264 ± 23.9 and 384 ± 12.2, respectively, after treatment with KuA for 24 h, the cell numbers penetrated the transwell membrane were decreased significantly in a dose-dependent manner, compared to the untreated GBM cells (Fig. 4E–H, *p* < 0.01). These results demonstrate that KuA suppressed invasion of U251 and WJ1 cells.

### KuA suppressed GBM growth *in vivo*

**Figure 5:**
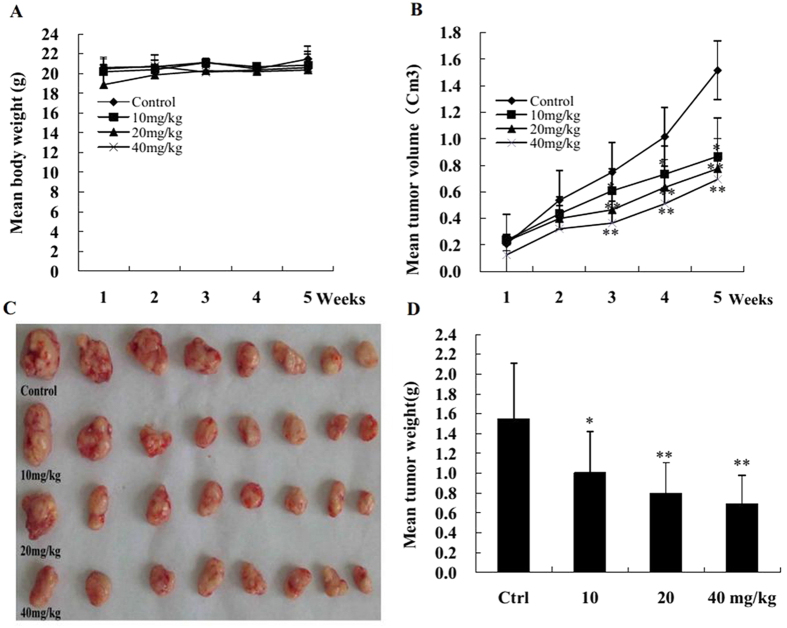
KuA inhibits human glioblastoma growth initiated from WJ1 cells *in vivo*. Mice were inoculated with 2 × 10^6^ human GBM cells (WJ1) each. One week after cell injection, three test group mice were administrated with 10, 20 and 40 mg/kg of KuA i.p. 5 times weekly for 4 weeks respectively. The control mice were injected with the same volume of solvent. Results of body weight, tumor volume and tumor weight are mean values ± SEM, *P < 0.05, **P < 0.01. (**A**) Body weight changes of each group after KuA treatment. (**B**) Tumor volume changes of each group after KuA treatment. (**C**) Tumor masses of each group. (**D**) The histogram shows that there was a significant difference of mean tumor weight of each KuA treated group compared with the control.

The tumor growth inhibition effect of KuA on GBM generated from WJ1 is shown in Fig. 5. KuA slowed down GBM growth significantly (Fig. 5B, *p* < 0.01). The mean tumor weight of the control mice was 1.55 ± 1.12 g and those of the mice treated with 10, 20, and 40 mg/kg of KuA were 1.0 ± 0.83 g, 0.79 ± 0.63 g and 0.69 ± 0.28 g, respectively (Fig. 5C,D).Tumor inhibitory rates were 35.2% (*p* < 0.05), 48.8% (*p* < 0.01) and 55.3%, (*p* < 0.01), respectively (Fig. 5D). No difference of body weight increase between the control and treated animals were observed (Fig. 5A). Together, this result proved that KuA inhibited GBM cell growth *in vivo*.

### KuA affected apoptosis and cell migration associated protein expressions *in vitro* and *in vivo*

**Figure 6:**
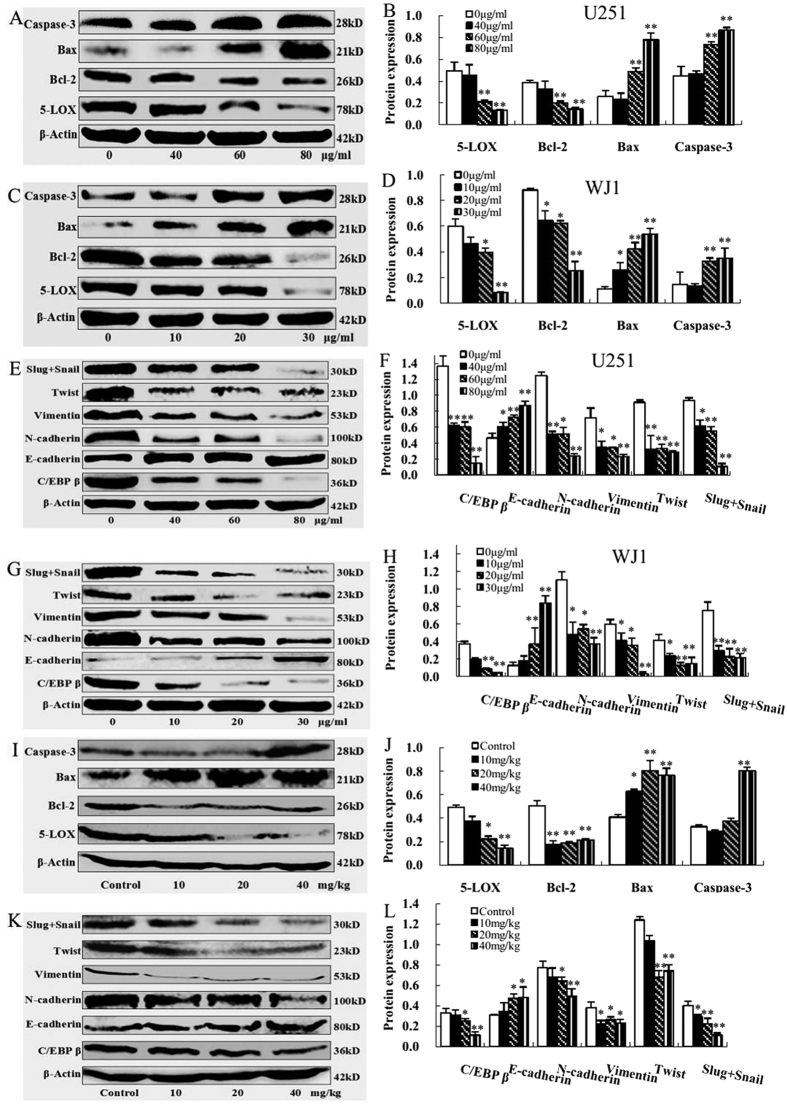
5-LOX, apoptotic protein, C/EBPβ and EMT associated protein expressions in KuA treated human glioblastoma cells and tumor tissues. 5 Proteins of both the untreated and KuA treated GBM cells (U251 and WJ1) and tumor tissues (WJ1) were prepared and separated on 10–15% SDS-PAGE. 5-LOX, apoptotic protein, C/EBPβ and EMT associated protein expressions were analyzed with Western blotting. Results are mean values ± SEM of three independent experiments, *P < 0.05, **P < 0.01. (**A**) Western blot analysis of 5-LOX and apoptotic protein expressions in KuA treated U251 cells. (**B**) The histogram shows that there was significant decrease of 5-LOX and Bcl-2 and increase of Bax and active caspaes-3 expressions in a dose dependent manner after KuA treatment in U251 cells. (**C**) Western blot analysis of 5-LOX and apoptotic protein expressions in KuA treated WJ1 cells. (**D**) The histogram shows that there was significant decrease of 5-LOX and Bcl-2 and increase of Bax and active caspaes-3 expressions in a dose dependent manner after KuA treatment in WJ1 cells. (**E**) Western blot analysis of C/EBPβ and EMT associated protein expressions in KuA treated U251 cells. (**F**) The histogram shows that there was significant decrease of C/EBPβ, N-cadherin, vimentin, twist and snail+slug and increase of E-cadherin expressions in a dose dependent manner after KuA treatment in U251 cells. (**G**) Western blot analysis of C/EBPβ and EMT associated protein expressions in KuA treated WJ1 cells. (**H**) The histogram shows that there was significant decrease of C/EBPβ, N-cadherin, vimentin, twist and snail + slug and increase of E-cadherin expressions in a dose dependent manner after KuA treatment in WJ1 cells. (**I**) Western blot analysis of 5-LOX and apoptotic protein expressions in KuA treated GBM tumor tissues. (**J**) The histogram shows that there was significant decrease of 5-LOX and Bcl-2 and increase of Bax and active caspaes-3 expressions in a dose dependent manner in GBM tumor tissues. (**K**) Western blot analysis of C/EBPβ and EMT associated protein expressions in KuA treated GBM tumor tissues. (**L**) The histogram shows that there was significant decrease of C/EBPβ, N-cadherin, vimentin, twist and snail + slug and increase of E-cadherin expressions in a dose dependent manner in KuA treated GBM tumor tissues.

To explore the potential molecular mechanisms underlying the growth and migration inhibition effect of KuA on human GBM cells *in vitro* and *in vivo*, apoptosis and cell migration related protein expressions in KuA treated U251cells, WJ1 cells and tumor tissues initiated from WJ1 cells were evaluated by Western blotting. Expressions of 5-LOX and Bcl-2 proteins were decreased significantly; expressions of Bax and active caspase-3 were increased significantly in KuA treated U251cells, WJ1 cells (Fig. 6A–D, *p* < 0.01) and tumor tissues (Fig. 6I,J, *p* < 0.01); the expressions of C/EBPβ, N-cadherin, vimentin, twist and snail+slug were decreased significantly, while the expression of E-cadherin was increased significantly in KuA treated U251cells, WJ1 cells (Fig. 6E–H, *p* < 0.01) and tumor tissues (Fig. 6K,L, *p* < 0.01) compared with the controls. Together, these results suggest that KuA inhibited human GBM cell growth through apoptosis induction by decreasing the expressions of 5-Lipoxygenase and Bcl-2, increasing expressions of Bax and active caspaes-3; it suppressed GBM cell migration through epithelial-mesenchymal transition attenuation by downregulating the expressions of C/EBPβ, N-cadherin, vimentin, twist and snail+slug, and upregulating the expression of E-cadherin.

## Discussion

Glioblastoma (GBM) is the most common malignant primary brain tumor with devastating proliferative and invasive characteristics; its aggressive growth pattern led GBM patients face a poor prognosis even after having received the best available treatment modalities^1^,^2^,^3^,^4^,^28^,^29^. However, the distinct biological behavior of aggressive proliferation and invasive growth of GBM might be the unexplored therapeutic targets for treatment of this fatal tumor^5^. Thus, the discovery of new and specific chemotherapeutic agents inhibiting proliferation, migration and invasion of GBM cells are regarded as effective strategies to research and develop new drugs for GBM therapeutics^10^,^11^,^12^,^13^,^14^,^28^.

Kukoamine A (KuA) is spermine alkaloid derived from a traditional Chinese herb medicine, *Cortex lycii radicis. It has* hypotensive, hypoglycemic, antipyretic, antioxidant, anti-inflammatory, soybean lipoxygenase inhibition and neuroprotective activities^18^,^19^, but the anticancer activity of KuA and its underlying mechanism are unknown.

In this experimental study, human normal liver cells (LO2), rat glioma cells (C6), and human GBM cells were treated with KuA *in vitro*, the proliferation and cloning efficiency of human GBM cells were inhibited in a time- and dose-dependent manner, Little effect on human normal liver cells (LO2), were observed; human GBM cells are more sensitive to KuA than rat glioma cells (C6). After KuA treatment, apoptotic cells were increased dose-dependently; furthermore, the motility of KuA treated GBM cells (U251 and WJ1) were decreased significantly; *In vivo* experiment, KuA slowed down the tumor growth initiated from GBM cells (WJ1) and reduced the mean tumor weight significantly. These findings suggest that KuA might have potential growth and migration inhibition effect on human GBM cells i*n vitro* and inhibits GBM growth *in vivo*.

To elucidate the molecular mechanisms of the growth and migration inhibition effects of KuA on human GBM cells *in vitro* and *in vivo*, the expressions of 5-Lipoxygenase, apoptotic proteins, CCAAT/enhancer binding protein β (C/EBPβ) and EMT associated proteins in KuA treated GBM cells and tumor tissues were analyzed with Western blotting.

5-lipoxygenase (5-LOX) is an enzyme in charge of the metabolism of arachidonic acid to leukotrienes^27^. It plays an important role in carcinogenesis and tumor growth^30^. Increasing evidence indicated that 5-LOX is involved in the progression of different types of cancer, including glioblastoma. It promotes cancer cell survival, proliferation, migration, invasion, metastasis, and activation of anti-apoptotic signaling parhways^21^,^22^,^24^,^31^. 5-LOX overexpression was associated with poor prognosis of glioblastoma patients^21^,^22^. Inhibition of 5-LOX activity could reduce the proliferation activity of cancer cells^31^, and induce glioblastoma cell apoptotic death^24^,^25^,^26^,^32^. Besides triggering apoptosis, 5-LOX was also involved in promotion of epithelial-mesenchymal transition (EMT) of cancer cells, abrogation of 5-LOX expression led to reduce featured molecular markers of EMT, including inactivation of E-cadherin and activation of snail, and cancer cell invasion^33^.

In this experimental study, KuA induced GBM cells apoptotic death dose-dependently, up-regulated the protein expressions of Bax and caspase-3 and down-regulated the expressions of Bcl-2 significantly in KuA treated glioblastoma cells and tumor tissues dose-dependently. It was suggested that KuA might have effects on activating intrinsic mitochondrial apoptotic pathway in glioblastoma cells, induces them caspase-dependent intrinsic apoptosis by 5-LOX inhibition.

Cancer cell migration and invasion resulted in the diffuse and invasive growth of glioblastoma, which makes it difficult to eradicate GBM for conventional therapeutics^3^,^4^,^5^. A mesenchymal phenotype is the biological character of GBM, GBM cells often obtain the ability to migrate, invade and metastasize through epithelial-to-mesenchymal transition (EMT)^1^,^34^,^35^,^36^.

CCAAT/enhancer binding protein β (C/EBPβ) plays an important role in GBM cell growth, migration and invasion through EMT regulation, reduction of its expression inhibits the growth and invasion of glioblastoma cells^34^,^37^,^38^. In epithelial to mesenchymal transition, E-cadherin is a marker of epithelial phenotype, while, N-cadherin and vimentin are the markers for mesenchymal phenotype^39^; Twist, slug and snail are the master regulators of the epithelial-mesenchymal transition^40^,^41^, they worked coordinately in regulation of GBM cell migration, invasion and metastasis through induction of EMT. Inhibition of the expressions of twist, slug and snail, the proliferation, migration, and invasion of glioblastoma cells were significantly suppressed^41^,^42^,^43^.

In present experiment, KuA inhibited GBM cell migration and invasion, and tumor growth dose-dependently. After KuA treatment, protein expressions of C/EBPβ, N-cadherin, vimentin, twist, slug and snail were downregulated significantly, while the expression of E-cadherin was upregulated significantly in KuA treated GBM cells and tumor tissues compared with the controls. It is suggested that EMT was be abated in GBM cells by KuA exposure, which might be associated with downregulating expression of C/EBPβ.

Based on the findings of this experimental study, it could be speculated that KuA treatment inhibited the expressions of 5-LOX and C/EBPβ in human GBM cells, which resulted in apoptosis and EMT attenuation of GBM cells, thereby inhibited human GBM cell growth and migration *in vitro* and *in vivo*.

**Figure 7:**
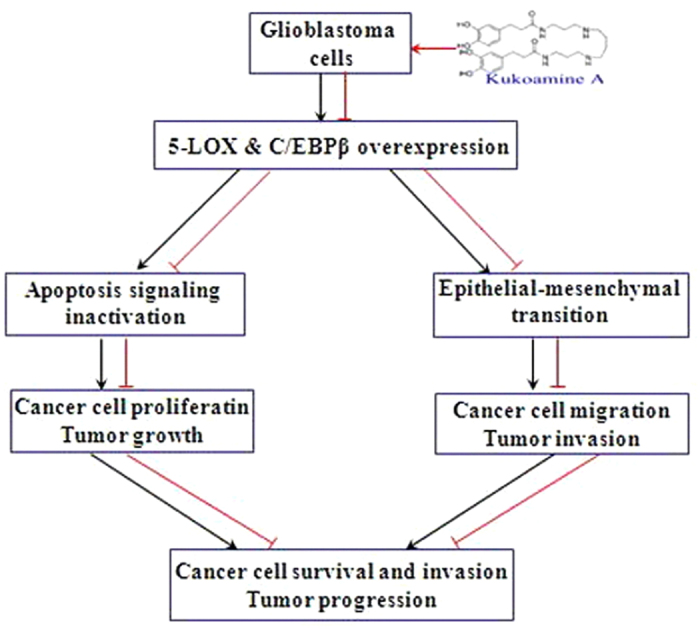
A schematic overview of human GBM cells response to KuA and its mechanisms of activity.

Taken together, Kukoamine A has the potential to inhibit human glioblastoma cell growth and migration *in vitro* and *in vivo* through apoptosis induction and epithelial-mesenchymal transition attenuation mediated by downregulating expressions of 5-LOX and C/EBPβ (Fig. 7); it might serve as an effective candidate agent for the treatment and/or prevention of human glioblastoma, and deserve to be investigated further.

## Materials and Methods

### Reagents and antibodies

DMEM medium and fetal bovine serum (FBS) were purchased from Gibco/BRL Invitrogen (Shanghai, China), 3-(4,5-dimethylthiazol-2-yl)-2,5-diphenyltetrazolium bromide (MTT), DMSO, and other chemicals and reagents were purchased from Sigma–Aldrich (Shanghai, China). Kukoamine A (KuA) was purchased from Chengdu Biopurify Phytochemicals Ltd. (Chengdu, China, purity ≥98% HPLC).

Rabbit anti-β-actin, 5-LOX, Bcl-2, Bax, Caspase-3, C/EBP β, E-cadherin, N-cadherin, Vimentin, Twist, Snail+slug primary antibodies and peroxidase-conjugated goat anti-rabbit IgG (H+L) secondary antibody were purchased from Beijing Biosynthesis Biotechnology Co., Ltd. (Beijing, China).

### Cell line and culture

Human normal liver cells (LO2), rat glioma cells (C6), and human glioblastoma (GBM) cell lines (U251) were obtained from China Center for Type Culture Collection (Wuhan, China), human GBM cell line (WJ1) was established, characterized, and kept in our laboratory^44^. The cells were cultured with DMEM medium containing 10% fetal bovine serum, penicillin (100 U/mL), and streptomycin (100 μg/mL), and maintained in a humidified atmosphere of 5% CO2 at 37 °C.

### Cytotoxicity assay (MTT)

MTT assay^45^ was used to test proliferation inhibition effect of kukoamine A (KuA) on normal cells and GBM cells. Briefly, cells (1.5 × 10^3^ cells/well) in 100 μl of medium were seeded in 96-well plates, after overnight incubation, different concentrations of KuA (10, 20, 40, 60 and 80 μg/ml) were added, 3 wells were included in each concentration. After treatment with KuA for 1, 2, 3, 4, and 5 days, 20 μL MTT (pH 4.7) was added to each well, cultivated for another 4 h, and then 100 μL of DMSO was added to each well to dissolve the formazan. Absorbance was measured at 570 nm, the effect of KuA on the viabilities of on normal cells and GBM cells were calculated by: (OD_570nm_ of drug-treated samples)/(OD_570nm_ of none treated samples) ×100%^46^. Three independent experiments were performed.

### Colony formation assay

GBM cells (U251 and WJ1) were seeded in 6 well plates at a density of 150 cells/well and 300 cells/well, respectively, After incubation overnight, different concentrations of KuA (5, 10 and 20 μg/ml) was added, and incubated for 12 days. The cells were fixed with methanol for 15 min and stained with 0.1% crystal violet for 10–20 min, the cell aggregates with >50 cells were scored as a colony. Colony forming efficiency was expressed as: Colony forming efficiency = (colony number of drug-treated cells/cell population) ×100%^47^. Three independent experiments were performed.

### AO/EB staining apoptotic cells

Both U251and WJ1 were seeded in 96-well plates (2000/well), and treated with different concentrations of KuA (40, 60 and 80 μg/ml for U251; 10, 20 and 30 μg/mL for WJ1) for 48 h respectively. The untreated and treated cells were stained with AO/EB dye mix (100 μg/mL acridine orange and 100 μg/mL ethidium bromide) for 2 min to detect apoptotic cells^48^. The stained cells were visualized immediately, images were captured using a Leica DMI400B inverted fluorescence microscope linked to a DFC340FX camera, more than 500 cells were counted for each sample. Three independent experiments were performed.

### Flow cytometry analysis of apoptotic cells

KuA induced apoptotic cells were detected using the Annexin V-FITC/PI apoptosis detection kit (Beyotime Biotech, Shanghai, China) according to manufacturer’s instructions. Briefly, GBM cells (U251 and WJ1) treated with 10–80 μg/mL of KuA for 48 h were harvested, washed twice in ice cold PBS and stained simultaneously with FITC-conjugated Annexin V and PI at room temperature for 15 min in the dark. The apoptotic cells were analyzed using a FACScalibur flow cytometer and Cell Quest Pro software (BD Biosciences, Shanghai, China). Three independent experiments were performed.

### Cell cycle analysis (FCM)

Cell cycle distribution was analyzed by flow cytometry. Briefly, 1 × 10^6^ cells were harvested from the control and GBM cells (U251, WJ1) treated with 5, 10, 20 μg/ml of KuA for 48 h, washed twice with PBS and fixed in 70% ice-cold ethanol for 1 h. The sample was then concentrated by removing ethanol and treated with 1% (v/v) Triton X-100 and 0.01% RNase for 10 min at 37 °C. Cellular DNA was stained with 0.05% propidium iodide for 20 min at 4 °C in darkness. Cell cycle distribution were analyzed with FCM (Cytomics^TM^ FC500, Beckman Coulter) and MultiCycle software package (Phoenix, USA). All data represents the results from three independent experiments.

### Wound healing assay

GBM cells (4 × 10^5^ cells/ml) were seeded in 12-well plates, incubated overnight and reached confluent monolayers for wounding. Wounds were made with a 10 μL pipette tip, various concentrations of KuA was added to monolayer GBM cells. Photographs were taken immediately (time 0) and 24 h after wounding for GBM cells under microscope (Nikon ECLIPSE Ti-U, Japan). The distances migrated by the monolayer cell to close the wounded area during this time course were measured using Image-Pro Plus 6.0 software. Results were expressed as a migration index—that is, the distance migrated by KuA treated cells relative to the distance migrated by the untreated cells^49^. Three independent experiments were performed.

### Cell invasion assay

Cell invasion assay was performed in 24-transwell chambers (Corning, Costar, USA), with 8 μm pores^49^. GBM cells were treated with different concentrations of KuA for 24 h, and harvested. 4 × 10^4^ cells in 200 μL serum-free medium were plated on the upper chamber, the lower chamber contained 500 μL of complete medium containing 15% FBS as a chemo-attractant. After 24 h incubation, cells on the upper surface of the membrane were scrubbed, and the cells that penetrated through the filter were fixed with methanol, stained with 0.1% crystal violet. Images were taken under microscope (Nikon ECLIPSE Ti-U, Japan), the penetrated cells in 5 non-overlapping random fields per well were counted. Three independent experiments were performed.

### GBM growth inhibition test *in vivo*

Animal care and experiments were conducted according to the guidelines approved by the Institutional Animal Care and Use Committee of Sichuan University. 32 five-week-old male nude mice (BALB/C-nu/nu) were inoculated with 2 × 10^6^ human GBM cells (WJ1). One week later, the tumor bearing mice were randomized into 4 groups and each group containing 8 mice, three test group mice were administrated with 10, 20 and 40 mg/kg of KuA i.p., 5 times weekly for 4 weeks, respectively; the control mice were injected with the same volume of solvent; During the experimental process, mice were weighted, and tumor volumes were measured every two days, tumor volumes were calculated using a standard formula (length × width^2^ × 0.5)^50^. At the end of experiment, the mice were sacrificed by carbon dioxide asphyxiation and dissected; the tumors were taken out and weighed. The tumor inhibitory rates were calculated using the formula: tumor inhibitory rate (%) = (mean tumor weight of the control group − mean tumor weight of the treated group) ÷mean tumor weight of the control group × 100%. All animals were maintained under standard conditions according to the guidelines of the Institutional Animal Care and Use Committee of Sichuan University.

### Western blot

Both the untreated and KuA treated GBM cells (U251 and WJ1) and tumor tissues (WJ1) were lysated with RIPA lysis buffer to extract proteins. The extracted proteins (30 μg) were separated on 10–15% SDS-PAGE and transferred onto PVDF membranes. The membranes were then blocked with 5% non-fat milk at room temperature for 1 h followed by incubation with corresponding primary antibodies (Beijing Biosynthesis Biotechnology Co., Ltd., Beijing, China), as indicated: rabbit anti-β-actin (1:1000), rabbit anti-5-LOX (1:250), rabbit anti-Bcl-2 (1:250), rabbit anti-Bax (1:200), rabbit anti-active caspase-3 (1:100), rabbit anti-C/EBPβ (1:400), rabbit anti-E-cadherin (1:250), rabbit anti-N-cadherin (1:250), rabbit anti-vimentin (1:400), rabbit anti-twist (1:100) and rabbit anti-snail+slug (1:100) overnight at 4 °C. Antibody recognition was detected with peroxidase-conjugated goat anti-rabbit IgG (H+L) secondary antibody (Zhongshan Goldenbridge Biotechnology Co., LTD, Beijing, China) used at 1:5000 dilutions, primary antibody binding were detected with Chemiluminescent HRP Substrate (Milipore Corporation, Billera, MA) and Western blotting analysis system (Universal Hood II, Bio-Rad), and normalized to β-actin and semi-quantified using the ChemiDocTM XRS (Bio-Rad).

### Statistical analysis

All values are expressed as means ± SD (standard deviation) and analyzed using the SPSS software (version 19.0) and analysis of variance (ANOVA), followed by Dunnett’s test for pairwise comparison. Statistical significance was defined as p < 0.05 for all tests.

## Additional Information

**How to cite this article**: Wang, Q. *et al*. Kukoamine A inhibits human glioblastoma cell growth and migration through apoptosis induction and epithelial-mesenchymal transition attenuation. *Sci. Rep.* **6**, 36543; doi: 10.1038/srep36543 (2016).

**Publisher’s note:** Springer Nature remains neutral with regard to jurisdictional claims in published maps and institutional affiliations.
